# Editorial: Mast cells in health, disease and forensic practice: a mystery not yet revealed

**DOI:** 10.3389/fmed.2023.1288565

**Published:** 2023-10-03

**Authors:** Stefano Bacci, Jared M. Brown, Domenico Ribatti

**Affiliations:** ^1^Department of Biology, Research Unit of Histology and Embryology, University of Florence, Florence, Italy; ^2^Skaggs School of Pharmacy and Pharmaceutical Sciences, University of Colorado Anschutz, Aurora, CO, United States; ^3^Department of Translational Biomedicine and Neuroscience, University of Bari Medical School, Bari, Italy

**Keywords:** allergy, bacterial infection, endometriosis, exosomes, Hodgkin's lymphoma, HSC, inflammation, mast cells

Ehrlich (1), identified mast cells (MCs) based on the metachromatic staining of their cytoplasmic granules in connective tissue. MCs are best known for their effector functions in allergic disorders. In recent years, however, MCs have been identified to be involved in a complex range of immune functions that go far beyond allergies and include the development of autoimmune disorders and peripheral tolerance other than the initiation and maintenance of adaptive and innate host responses. In addition, MCs are critical in numerous physiological and pathophysiological responses including cancer (2). It should also be added that the localization of these cells assumes importance in the forensic field, in particular in the aspects related to the recognition of the viability of the lesion (3). Therefore, understanding the significance of MCs and molecular mechanisms of immune and signaling events involving these cells can be of valuable help for therapeutic solutions to a variety of medical problems.

This editorial summarizes the contributions to the Frontiers Research Topic “*Mast cells in health, disease and forensic practice: a mystery not yet revealed*” appearing in Frontiers in Medicine section Translational Medicine.

## Review on general arguments

Dileepan et al. highlights the important role of MCs related to their ability to release a variety of effector molecules (thanks to the presence of surface and cytoplasmic receptors) that allow them to respond to various types of insults. Furthermore, a hypothesized involvement of MCs in the alterations induced by SARS-CoV-2 deepens the understanding of MCs in viral infections. Finally, the interaction between MCs and microglial cells in the brain further highlights their significance in neuroinflammation. Therefore, MCs act as a sensor for the fine regulation of an early response involving interactions with cells of systemic organs and nervous system.

## Review on specific arguments

Classical Hodgkin lymphoma (CHL) accounts for 10% of all lymphomas (4). Among histological subtypes, nodular sclerosis and mixed cellularity account for nearly 80% of all CHL cases. Since the increase in MCs in CHL appears to be greater in nodular sclerosis than in other subtypes of CHL and a correlation between the degree of angiogenesis and the number of MCs in CHL has been demonstrated, Ribatti et al. propose that MCs are considered possible new targets for disease treatment since novel therapies acting on these cell types inhibit angiogenesis and tissue remodeling and allow the secretion of cytotoxic cytokines such as Tumor Necrosis Factor.

Chronic rhinosinusitis with nasal polyposis (CRSwNP) a type 2 disease gives rise to polyps that block the sinuses and nostrils, leading to severe congestion, nasal discharge, pain, or pressure on the face, and reduction of smell and taste (5). Nasal cytology demonstrated that CRSwNP is characterized not only by eosinophilic but also MC inflammation and, in particular, the most severe forms of CRSwNP are characterized precisely by mixed eosinophilic-mast cell inflammation. Therefore, Gelardi et al. open the possibility for new therapies aimed at reducing both eosinophil and MC infiltration.

## Research articles on specific topics

### About the origin of MCs

Recent advances in developmental immunology have revealed a hematopoietic stem cell (HSC)-independent origin for various cell types, including MCs. It is now established that the physiological production of MCs in the aorta-gonad-mesonephros (AGM) region is mostly completed before birth. However, if the AGM region represents an important site of MC generation during ontogeny, it is unknown whether the first HSC emerging in the AGM or fetal liver (FL) possess the potential to regenerate MCs. Yoshimoto et al. demonstrated through sophisticated experiments that the first HSCs that emerge in the AGM and infiltrate the first FL can produce MCs, but only during a short time window.

### About the role of MCs in endometriosis

The influence of estrogen on MC function has also been recognized as a potential factor in endometriosis (6). McCallion et al. demonstrates a significant increase in the number of MCs within the endometriotic lesions compared to the corresponding eutopic endometrium of the same patients. Furthermore, an increase in SCF (essential for MC development) was found in endometriotic lesions while genetic analysis revealed a microenvironment favoring MC recruitment and subsequent differentiation. Cocultures of endometriotic epithelial cells and endometrial stromal cells incubated with MC-conditioned media showed a significant increase in the production of proinflammatory cytokines by these cells. Endometriotic lesions produced in C57BL/6 mice, confirm essentially previous findings in human. These results suggest that endometriotic lesions provide a microenvironment necessary for MC recruitment and differentiation in which the same cells release pro-inflammatory mediators that contribute to disease progression.

### About the role of MCs exosomes

Savages et al. using primary human lung fibroblasts (HLF), demonstrate that isolated and labeled exosomes from the human mast cell line as well as TGF-β have an additive effect on collagen synthesis in HLF and this fact could represent a unique paradigm for understanding fibrosis. The authors state that these findings provide rationale for targeting multiple fibrogenic pathways in the management of pulmonary fibrosis and for the use of MC exosomes as a biomarker for the prognostic and diagnostic management of evolving fibrotic lung disease as reported in patients with severe COVID-19.

## Conclusions

This topic was the occasion for a meeting of expert scientists, coming from different schools around the world, whose research field is aimed toward understanding MC biology. The reviews have proved to be punctual in the clarity and exposition, the research articles have proposed new methods and field of research that will certainly be deepened in the near future.

In conclusion, from this topic emerges the idea that the issue of MCs biology is extremely important and therefore the idea that the “mystery” of MCs is far from solved is confirmed.

## Author contributions

SB: Writing—review and editing. DR: Writing—review and editing. JB: Writing—review and editing.
